# AEG-1 aggravates inflammation via promoting NALP3 inflammasome formation in murine endometriosis lesions

**DOI:** 10.1080/19768354.2019.1691052

**Published:** 2019-11-21

**Authors:** Juan Zhao, Wei Ma, Weizhi Chen, Jie Gao, Chunling Li, Yahong Tong, Qin Zhou, Xiuling Zhao, Menghua Wang, Huan Xiao, Yanrong Jin

**Affiliations:** Department of Obstetrics and Gynecology, Beijing Luhe Hospital, Capital Medical University, Beijing, People’s Republic of China

**Keywords:** Endometriosis, astrocyte elevated gene-1, inflammation, nucleotide-binding oligomerization domain-like receptor 3, suppressor of cytokine signaling1

## Abstract

Endometriosis (EMs) is one of the most common gynaecological diseases in women of childbearing age. Astrocyte elevated gene-1 (AEG-1) is associated with the invasion, migration, apoptosis and prognosis of various cancers. However, the roles of AEG-1 in EMs and its corresponding molecular mechanism are still unknown. In this study, animal models of EMs were established and mice were divided into two groups (*n* = 10): Sham group and EMs group. The EMs cells were isolated from EMs model. The AEG-1 gene was knocked down by shRNA, while the SOCS1 gene was knocked down by siRNA. Histological changes, AEG-1 expression in tissues and inflammatory factors level were detected by H&E staining, immunohistochemistry and ELISA, respectively. RT-qPCR and western blotting were used to determine the expression level of related proteins. The present study found AEG-1 was up-regulated in the EMs model. Enhanced AEG-1 promoted inflammatory cell infiltration, and elevated the levels of IL-1β, IL-6, and TNF-α in EM group (*p* < 0.05). Besides, AEG-1 overexpression promoted the expression of NALP3, ASC and Cleaved-caspase-1, while decreased SOCS1 level (*p* < 0.05). Decrease of SOCS1 further promoted the formation of NALP3 inflammasome. The inhibitory effect of AEG-1 on SOCS1 was weakened after the addition of MG-132 (*p* < 0.01). Furthermore, silencing AEG-1 alone increased SOCS1 level, decreased the levels of inflammatory cytokines, thereby inhibited the formation of NALP3 inflammasome. All these results demonstrated that AEG-1 aggravated inflammation via promoting NALP3 inflammasome formation in murine endometriosis lesions.

## Introduction

Endometriosis (EMs) is one of the most common gynaecological diseases in women of childbearing age. It is characterized by the migration and growth of endometrial tissue outside the uterus, forming an ectopic lesion (Parasar et al. 2017). About 10% to 15% of women of childbearing age suffer from EMs, and its incidence has increased significantly in recent years (Bulun 2009). The main treatment methods are surgery and drug treatment. Among them, drug treatment can only treat symptomatic disease, while the surgical treatment has a high recurrence rate (Vercellini et al. 2014). However, the etiology of endometriosis has not been fully elucidated. Recent studies have shown that the intra-abdominal inflammatory environment and immune abnormalities are closely related to ectopic endometrial hyperplasia (Dai et al. 2019; Dull et al. 2019). Therefore, exploring the potential molecular mechanisms of EMs is important for the diagnosis and treatment of EMs.

Astrocyte elevated gene-1 (AEG-1), is originally discovered in human fetal astrocytes and regulated by HIV-1 or TNF-α (Su et al. 2002; Kang et al. 2005). Numerous studies have manifestedAEG-1 is involved in the motility, apoptosis and prognosis of various cancer cells, such as gastric cancer (Yu et al. 2018a), thyroid cancer (Fang et al. 2018), pancreatic cancer cells (Yang and Song 2018), hepatocellular carcinogenesis (Robertson et al. 2018) and ovarian cancer (Yu et al. 2018b). Besides, AGE-1 is also associated with diseases other than tumors. Zhao et al. found that the of AEG-1 expression was up-regulated in streptozocin-induced diabetic cardiomyopathy mice (Zhao et al. 2018) Furthermore, Peng et al. reported that AEG-1 low-expression could suppresses NLRP3 expression and promote caspase-1 activation, which further reduced the levels of IL-1β and IL-18. while AEG-1 overexpression deteriorated LPS-induced mucosal barrier injury and activation of NLRP3 inflammasome (Peng et al. 2019). At present, the roles and potential molecular mechanism of AGE-1 in EMs have not been reported.

Nucleotide-binding oligomerization domain-like receptor 3 (NALP3) is an important inflammatory mediator in inflammatory response. NALP3 inflammasome is composed of NALP3, apoptosis-related dot-like protein (ASC) and effector protein caspase-1 (Mezzasoma et al. 2017). Study found that NALP3 inflammasome could activate caspase-1, and further mediate the maturation and secretion of IL-1β, and regulate the body's inflammatory immune response (Zhang et al. 2018). SOCS-1 is a member of the SOCS family that is involved in the progression of inflammation and cancers. SOCS-1 could inhibit TLR signaling and NF-κB signaling pathways (Mansell et al. 2006; Strebovsky et al. 2011). The present study aimed to investigate the roles of AGE-1 and its potential molecular mechanism in EMs. The study found that AEG-1 aggravated inflammation via promoting NALP3 inflammasome formation in murine endometriosis lesions.

## Material and methods

### Animal model and group

Animal experiments are undertaken according to the NIH Guide for the Care and Use of Laboratory Animals and are approved by Beijing Luhe Hospital (No.2018-LHKY-013-03). Twenty C57BL/6 mice (female, 7–8 weeks old, SPF grade, 18–21 g) are obtained from the Animal Center of Beijing Luhe Hospital. The mice were fed with standard light cycle (12 h white light, 12 h night), indoor temperature 18-25°C, humidity 50% ∼ 65%, ammonia concentration ≤14 mg/m^3^, standard feed and water, and anesthetized by intraperitoneal injection of 5% chloral hydrate 0.1 ml/10 g. The animal model was constructed as previously reported (Rossi et al. 2000). After successful modeling, mice were divided into two groups (*n* = 10): Sham group and EMs group.

## Hematoxylin–eosin (H&E) staining

The endometrial tissues of the Sham group and the EMs group were fixed in 4% formaldehyde for 72 h. The tissue was decalcified in 20% ethylenediaminetetraacetic acid, dehydrated in a gradient of ethanol, and fixed in paraffin. Then, the tissues were cut into pieces and stained with hematoxylin–eosin (H&E) staining, respectively. Morphological changes were observed with an optical microscope (BX51; Olympus Corp., Tokyo, Japan).

## Reverse transcription quantitative real-time PCR (RT-qPCR)

Total RNAs were isolated with TRNzol Universal Reagent (TIANGEN BIOTECH, Beijing, China), while RT-qPCR reaction was performed with Quant One Step RT-qPCR Kit (Probe, TIANGEN BIOTECH, Beijing, China) by using the Bio-Rad CFX96 PCR System (Bio-Rad, CA, USA). All primers were as follows: IL-1β, forward 5′-CTAGGCATTGACCAGAATGAC-3′; reverse 5′-GATTCATTACGGCATTGGC-3′. IL-6, forward 5′-TCAACGGTAACGATGCACG-3′; reverse 5′-GGTCAATTGCGTAACGTGT-3′. TNF-α, forward 5′-GGTACGATAACGTCGATGC-3′; reverse 5′-CCTAGCGAACGTGGAACTGC-3′. AEG-1, forward 5′-GGGTCATAAGGAAACGGTCC-3′; reverse 5′TAGGTTACGCCCGTGGTACGTT-3′. SOCS1, forward 5′-CTGAAACTGCCCTGACCATG-3′; reverse 5′-ATGCCCTGACGGTACGGCATT-3′. GAPDH, forward 5′-CATTTGAAACGTGCATGCA-3′; reverse 5′-TGCCTACGTGCAATGGTCGCG-3′. (TransGen Biotech, Beijing, China). CT value was normalized to GAPDH and calculated with the 2^−ΔΔCt^.

## Enzyme-Linked Immuno Sorbent assay (ELISA)

Serum were collected and stored at −80°C for analysis. Cytokines including IL-1β, IL-6, and TNF-α were monitored with ELISA kits in accordance to the manufacturer’s instructions (Bio-Swamp, Shanghai, China). A microplate reader (BioTek Epoch, Winooski, VT, USA) was utilized to examine the OD values at 450 nm. Quantitative analysis of cytokines were monitored according to the standard curve.

## Western blotting

Proteins were isolated from the endometrial tissues or EMs cells and determined with BCA protein assay kit (Pierce, USA). Samples were separated by 12% SDS-PAGE and transferred to PVDF membranes (EMD Millipore, Billerica, MA, USA). After sealing with 5% BSA for 1 h at 25 ˚C, the samples were probed with corresponding primary antibodies against GAPDH (#5174, 1:1000, Cell Signaling Technology, Massachusetts, USA), AEG-1 (#14065, 1:1000, CST, Massachusetts, USA), NALP3 (#15101, 1:1000, CST, Massachusetts, USA), ASC (#67824, 1:1000, CST, Massachusetts, USA), Cleaved-caspased-1 (#4199, 1:1000, CST, Massachusetts, USA), SOCS1 (#3950, 1:1000, CST, Massachusetts, USA) at 4˚C overnight. The next day, samples were probed with anti-rabbit IgG (H + L) (#14708, 1:1000, CST, Massachusetts, USA) for 1.5 h at 25 ˚C, and visualized using enhanced chemiluminescence reagents (Pierce, USA).

## Immunohistochemistry

The embedded paraffin sections were separated by xylene and rehydrated in different concentrations of ethanol. The sections were then incubated in 3% H_2_O_2_and blocked with AEG-1 antibody for 1 h (#14065, 1: 150, CST, Massachusetts, USA) overnight at 4°C. The corresponding secondary (#8112, CST, Massachusetts, USA) antibody was added for 1 h at 25°C. These images were observed under an optical microscope.

## Isolation and culture of primary cells

Isolation of intimal cells was performed as previously reported (Reis et al. 2001). Intima fragments (1 mm × 1 mm) and 0.2% type I collagenase was mixed at a ratio of 2: 1–3: 1, digested in a 37°C, 5% CO_2_ incubator for 70 min, the supernatant was discarded, and dNaseI was added in a ratio of 1: 1 at 37°C with 5% CO_2_ for 20–30 min. Complete culture solution was used to terminate digestion, mix by pipetting, and purify the cells by differential adherence method. The primary cells were cultured in DMEM/F12 at 37°C with 5% CO_2_.

## Transfection

Overexpression vectors (LV-AEG-1, LV-NC), and knockout vectors (sh1#-AEG-1, sh2#-AEG-1, siNC, siSOCS1-1#, siSOCS1-2#) were purchased from GeneChem (Shanghai, China). EMs cells were transfected with these vectors with 5 μg/mL polybrene (GeneChem). After infection for 48 h, the transfection efficacy was determined by RT-qPCR. After 2 days of infection, the cells were screened with 2 μg/mL puromycin for 2 weeks, and stable cell line was established. Transfections in this study were carried out by Lipofectamine2000 transfection reagent in accordance with the manufacturer's protocol (Invitrogen, Grand Island, NY, USA)

## Statistic analysis

All data are shown as mean ± standard deviation and repeated at least three times. One-way analysis of variance was applied to make statistical comparisons between the different groups. SPSS 13.0 (SPSS, Inc., Chicago, IL, USA) was used for the Bonferroni post hoc test. *p* < 0.05 was considered to represent a statistically significant difference.

## Results

### AEG-1 is up-regulated in the EMs model

**Figure 1.**
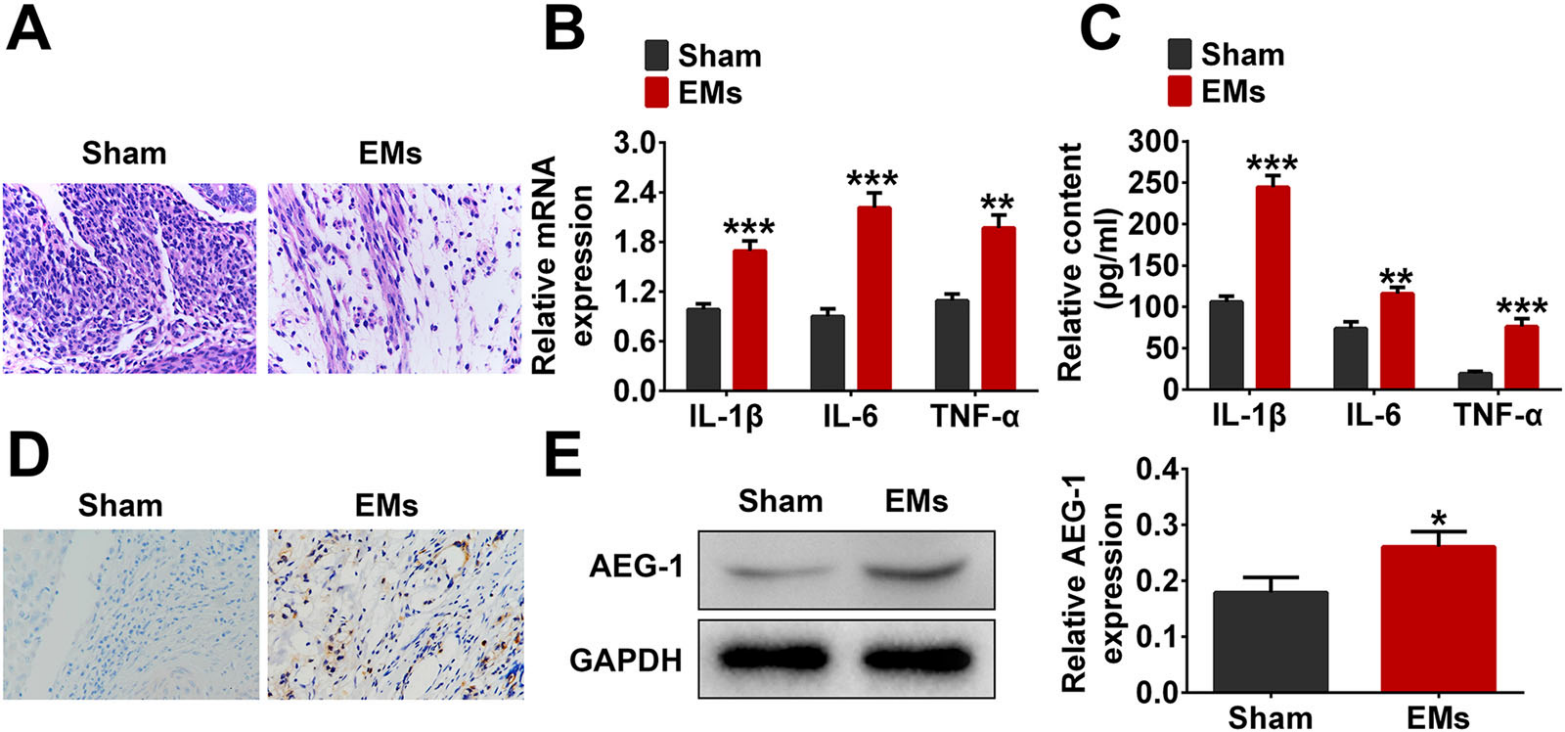
AEG-1 is up-regulated in the EMs model. The mice were divided into two group (*n* = 10): Sham group and EMs group. A. Histopathological changes detected by H&E staining. B. The expression levels of IL-1β, IL-6, and TNF-α were monitored by RT-qPCR. C. The contents of IL-1β, IL-6, and TNF-α were detected by ELISA. D. The expression of AEG-1 was monitored by immunohistochemistry. E. The protein level of AEG-1 was detected by western blotting (*p < 0.05, **p < 0.01, ***p < 0.001).

To explore AEG-1 expression and its effects on endometrial inflammatory response, H&E, RT-qPCR, immunohistochemistry, ELISA and western blotting were performed. As shown in Figure 1(A), a large amount of inflammatory cell infiltration occurred in the EMs group compared with the Sham group. ELISA and RT-qPCR assays showed that the levels of inflammatory cytokines (IL-1β, IL-6, and TNF-α) in EM group were significantly higher than those in the Sham group (Figure 1(B,C)). In addition, western blotting and immunohistochemistry showed that AEG-1 was highly expressed in endometrial tissues of EMs group compared with Sham group (Figure 1(D,E)). These results suggested that AEG-1 was up-regulated in the EMs model.

## AEG-1 knockdown suppressed inflammation by inhibiting the formation of NALP3 inflammasome in EMs cells

**Figure 2.**
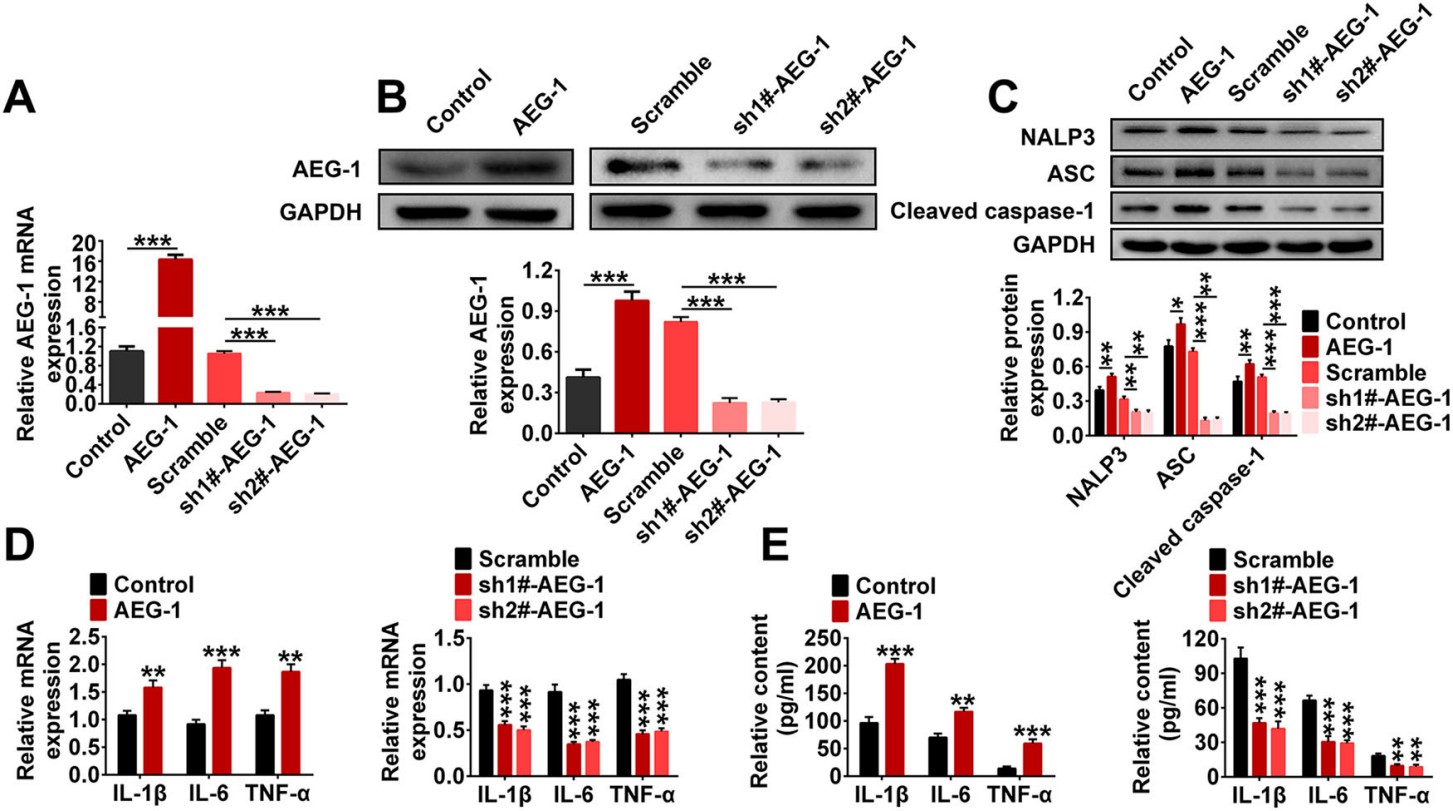
AEG-1 knockdown inhibited inflammation by inhibiting the formation of NALP3 inflammasome in EMs cells. EMs cells was transfected with AEG-1, Scramble, sh1#-AEG-1 and sh2#-AEG-1, respectively. A. The mRNA levels of AEG-1 was monitored by RT-qPCR. B. The protein level of AEG-1 was monitored by western blotting. C. The protein levels of NALP3, ASC and Cleaved-Caspase-1 were monitored by western blotting. D. The mRNA levels of IL-1β, IL-6, and TNF-α were monitored by RT-qPCR. E. The contents of IL-1β, IL-6, and TNF-α were monitored by ELISA (*p < 0.05, **p < 0.01, ***p < 0.001).

RT-qPCR, ELISA and western blotting were used to explore the interaction of AEG-1 with NALP3. As shown in Figure 2(A), AEG-1 level in the AEG-1 group were increased dramatically compared to the Control group. After shRNA interference, the levels of AEG-1 in the sh1#-AEG-1 and sh2#-AEG-1 group were significantly lower than that in the Scramble group. Similarly, western blotting analysis yielded consistent results (Figure 2(B)). In addition, western blotting results also showed that AEG-1 overexpression significantly elevated the levels of NALP3, ASC and Cleaved-caspase-1 compared to the Control group. After shRNA interference, The levels of these proteins decreased significantly (Figure 2(C)). Moreover, AEG-1 overexpression significantly increased the levels of inflammatory factors. AEG-1 knockdown had the opposite effects (Figure 2(D,E)). These results indicate that AEG-1 knockdown inhibited inflammation by inhibiting the formation of NALP3 inflammasome in EMs cells.

## Overexpression of SOCS1 inhibited inflammation by inhibiting the formation of NALP3 inflammasome in EMs cells

**Figure 3.**
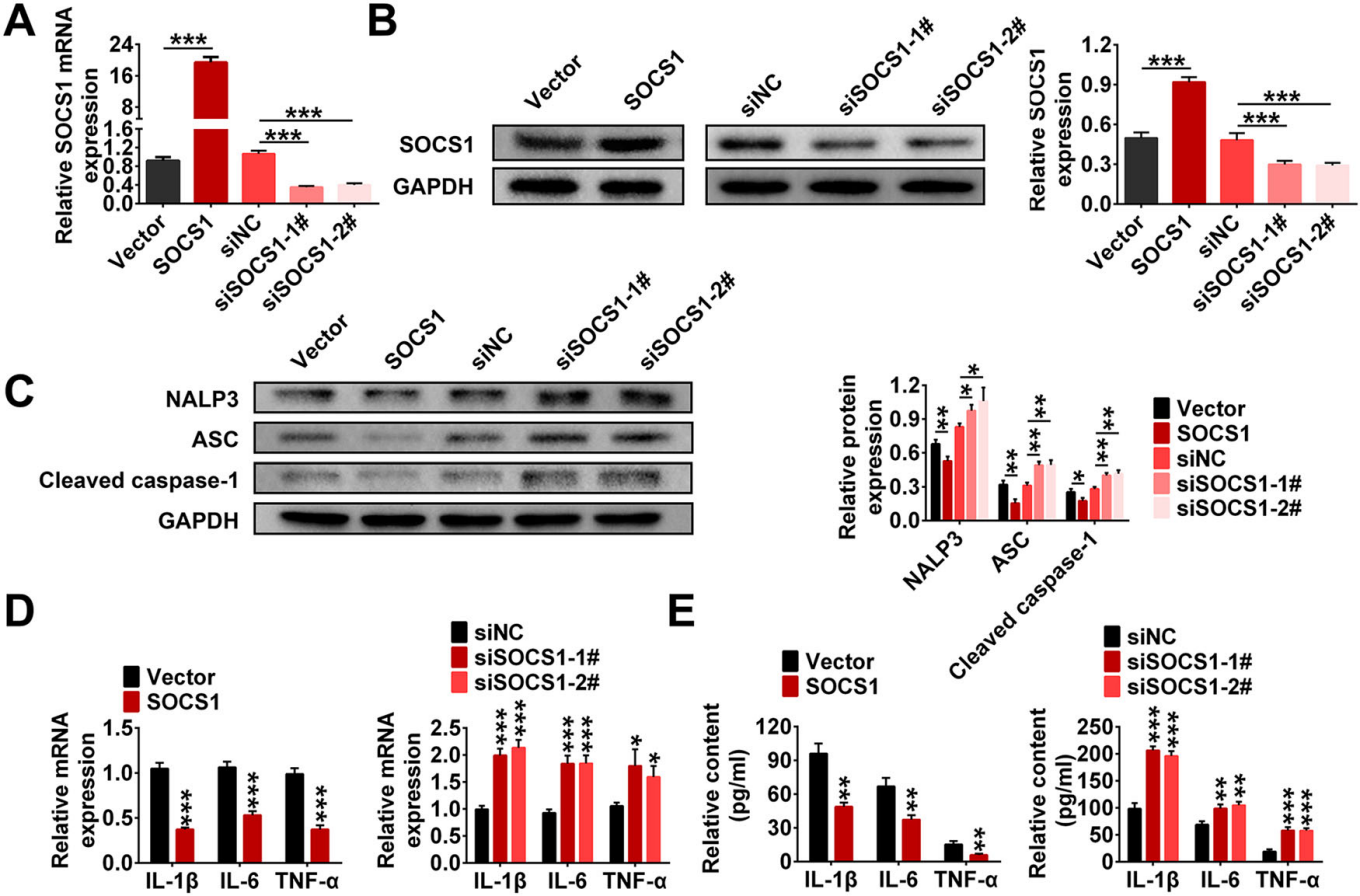
Overexpression of SOCS1 inhibited inflammation by inhibiting the formation of NALP3 inflammasome in EMs cells. EMs cells were transfected with Vector, SOCS1, siNC, siSOCS1-1#, siSOCS1-2#, respectively. A. The expression levels of SOCS1 was monitored by RT-qPCR. B. The protein level of SOCS1 was monitored by western blotting. C. The protein levels of NALP3, ASC and Cleaved-Caspase-1 were monitored by western blotting. D. The expression levels of IL-1β, IL-6, and TNF-α were monitored by RT-qPCR. E. The contents of IL-1β, IL-6, and TNF-α were detected by ELISA (*p < 0.05, **p < 0.01, ***p < 0.001).

This study also explored the effect of SOCS1 on the inflammation. As shown in Figure 3(A), the level of SOCS1 in the SOCS1 group was increased sharply compared to the Vector group. After siRNA interference, the SOCS1 levels in the siSOCS1-1# and siSOCS1-2# groups were significantly lower than that in the siNC group. Similarly, western blotting assay yielded consistent results (Figure 3(B)). In addition, western blotting also showed that SOCS1 overexpression significantly reduced the levels of NALP3, ASC and Cleaved-caspase-1 compared to the vector group. The levels of NALP3, ASC and Cleaved-caspase-1 were significantly increased after siRNA interference (Figure 3(C)). Moreover, SOCS1 overexpression significantly suppressed the levels of inflammatory factors. Knocking down SOCS1 had the opposite effects (Figure 3(D,E)). These results suggested that overexpression of SOCS1 inhibited inflammation by inhibiting the formation of NALP3 inflammasome in EMs cells.

## AEG-1 promoted the inflammation of EMs by reducing SOCS1 level to promote the formation of NALP3 inflammasome

**Figure 4.**
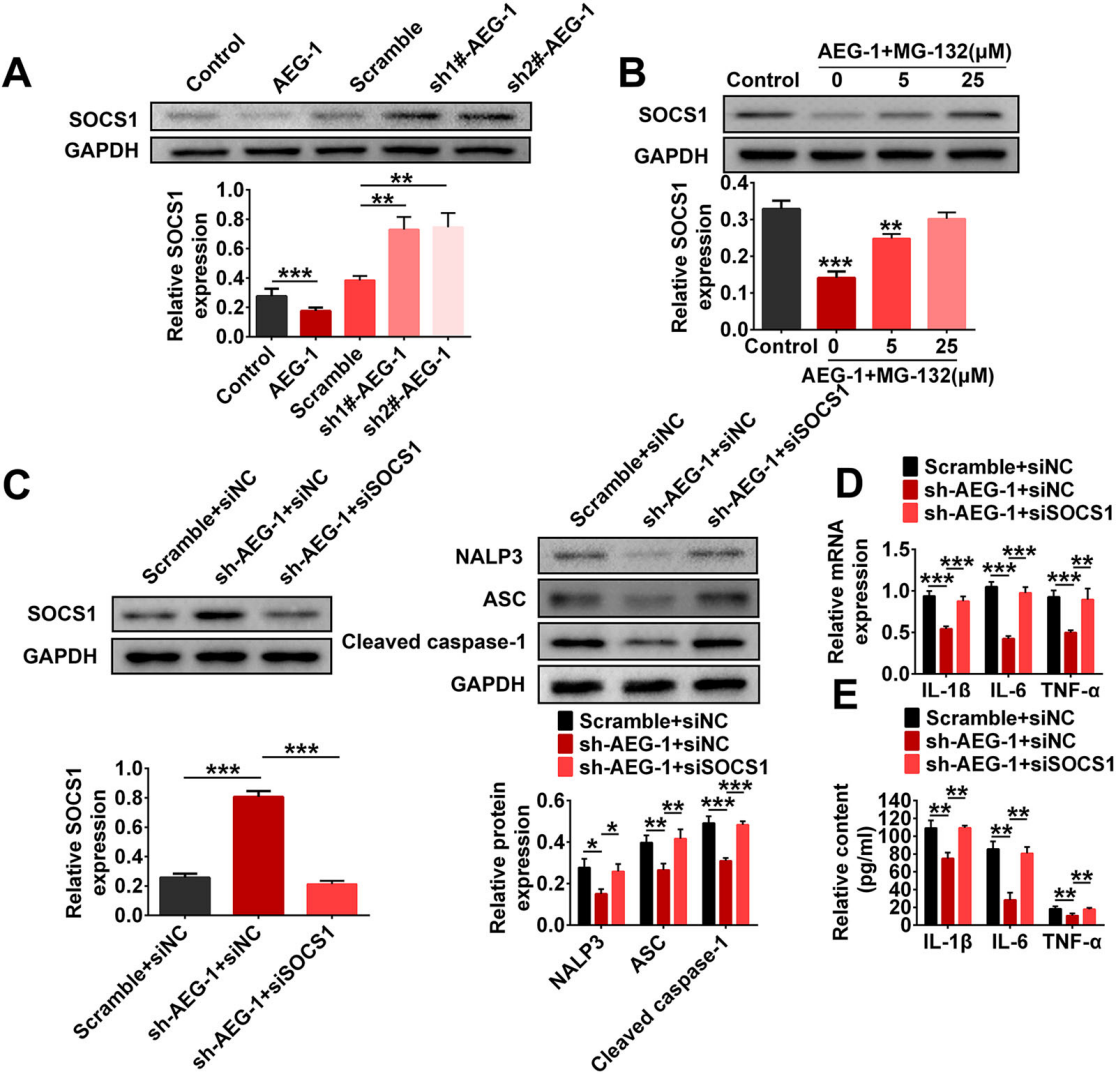
AEG-1 promoted the inflammation of EMs by reducing SOCS1 level to promote the formation of NALP3 inflammasome. A. EMs cells were transfected with AEG-1, Scramble, sh1#-AEG-1 and sh2#-AEG-1, respectively. The protein level of SOCS1 was monitored by western blotting. B. EMs cells transfected with AEG-1 were treated with different concentrations of the proteasome inhibitor MG-132 (0, 5, 25 μM). The protein level of SOCS1 was monitored by western blotting. C-E. EMs cells were transfected with Scramble + siNC, sh-AEG-1 + siNC or sh-AEG-1 + siSOCS1. C. The protein level of SOCS1 was monitored by western blotting. D. The mRNA levels of IL-1β, IL-6, and TNF-α were monitored by RT-qPCR. E. The contents of IL-1β, IL-6, and TNF-α were monitored by ELISA (*p < 0.05, **p < 0.01, ***p < 0.001).

To explore the molecular mechanism of AEG-1 response to inflammation, western blotting and ELISA were performed. As shown in Figure 4(A), the level of SOCSl in the AEG-1 group was significantly reduced compared to the Control group. After shRNA interference, the SOCS1 levels in the sh1#-AEG-1 and sh2#-AEG-1 group were significantly higher than in the control interference group. After the addition of different concentrations of the proteasome inhibitor MG-132 (0, 5, 25 μM), the inhibitory effects of AEG-1 on SOCS1 was weakened (Figure 4(B)). In addition, the western blotting results also showed that silencing AEG-1 alone increased SOCS1 level, decreased the levels of inflammatory cytokines, and inhibited the formation of NALP3 inflammasome, while simultaneous silencing of AEG-1 and SOCS1 had the opposite effects (Figure 4(C–E)). All the data showed that AEG-1 promoted the inflammation of EMs by reducing SOCS1 level to promote the formation of NALP3 inflammasome.

## Discussion

Past studies have shown that endometriosis is an estrogen-dependent and inflammatory disease associated with peripheral and central nervous system recovery in pain and infertility (Grandien 1996; Bulun 2009). Zhao et al. found that inhibition of estrogenic-associated inflammation could block the progression of EMs (Yuechao et al. 2015). The present study constructed an EMs animal model, and found that a large amount of inflammatory cell infiltration occurred in the EMs group compared with the Sham group. Besides, the levels of inflammatory cytokines (IL-1β, IL-6, and TNF-α) in EM group were significantly higher than those in the Sham group. These results elucidated that the progression of EMs was involved in the inflammation in mice.

AEG-1 is associated with the invasion, migration, apoptosis and prognosis of various cancers (Li et al. 2014). Besides, AEG-1 also plays an important role in the development of inflammation. As a member of SOCS family, SOCS-1 is the only one that can regulate NF-κB signaling pathway (Julia et al. 2011). Interestingly, AEG-1 could also interact with p65 to upregulate NF-κB pathway (Emdad et al. 2006). Li et al. found that AEG-1 accelerated gastric cancer progression by regulating proinflammatory TLR4/NF-kB signaling pathway (Li et al. 2014). The present study found that AEG-1 was upregulated in EMs mice. Enhanced AEG-1 significantly increased the levels of NALP3, ASC and cleaved-caspase-1, while decreased SOCS1 level. Moreover, AEG-1 overexpression significantly elevated the levels of inflammatory factors. All these data indicated that AEG-1 aggravates inflammation by decreasing SOCS1 level in EMs mice.

The previous studies have shown that inflammation is an important pathophysiological basis for EMs (Yilmaz and Bulun 2019). As an important inflammatory mediator in inflammatory response, NALP3 is an important component of inflammasome (Mezzasoma et al. 2017). NALP3 inflammasome can promote the maturation and production of inflammatory cytokines by activating caspase-1 (Sakai and Wada 2015). Recently, numerous evidences showed that inflammasome plays a key role in the occurrence and development of EMs (Jun et al. 2015; Bullon and Manuel Navarro 2017). This study explored the effect of NALP3 inflammatory inflammasome on the pathogenesis of EMs by establishing an EMs model. The results showed that overexpression of AEG-1 significantly reduced the levels of NALP3 and ASC, inhibited the activation of caspase-1, and inhibited the production of inflammatory cytokines.

## Conclusion

In conclusion, the study investigated the roles of AEG-1 and its potential molecular mechanism in EMs. The results showed that AEG-1 was up-regulated in the EMs model. Overexpression of AEG-1 significantly inhibited the formation of NALP3 inflammasome by downregulating SOCS1 level in EMs cells.
